# Sexual Hypochondriasis

**Published:** 1892-10

**Authors:** C. Evans Johnson

**Affiliations:** Atlanta, Ga.


### SEXUAL HYPOCHONDRIASIS.

BY C. EVANS JOHNSON, M. D., ATLANTA, GA.

Every one who does any work in the line of venereal diseases has had to deal to a greater or less extent with that peculiar class of cases termed sexual hypochondriacs, who apply complaining of an abnormal condition of things relative either to the appearance or the functions of their genital organs.

These patients are of two distinct classes. There are those who need only to be assured by you that both the appearance and the functions of these organs vary in different individuals and are yet normal, and those who refuse to accept all such statements—whose minds are really unsound in regard to this point, and who, unless something is done for them, and some pretense at treatment is made, will continue brooding over their supposed troubles, leading a life which is a burden alike to themselves and those around them, until their fitful career is ended within the walls of an asylum for the insane, or they fill a suicide's grave.

You will be consulted by men declaring that they have noticed a few herpetic vesicles appearing from time to time upon the penis and scrotum, that one testicle hangs lower than the other, that they have nocturnal emissions once or twice a month, that during an effort at sexual intercourse, ejaculation occurs before an entrance can be effected, or, possibly, they are suffering from impotence which will be found to be purely psychical.

But nocturnal emissions are the source of complaint of the greater number of sexual hypochondriacs, and will be ascribed by them to masturbation, which will be found to have been only moderately practiced by them in former years. Even men who have enjoyed a period of many years of vigorous sexual powers, and who, by reason of being left widowers or because of the ill-health of their wives are leading a life of continence, will ascribe an occasional nocturnal emission to their early indiscretions.

If such patients as these enumerated happen to be blessed with common sense, the majority can be convinced that the herpetic vesicles are of no consequence; that it is perfectly natural that one testicle should be the lower (which, in the greater number of instances, will be found to be the left, because of the fact that most men dress upon the right side, displacing the testicles to the left); that nocturnal emissions occur independent of the practice of masturbation, it frequently being found in subjects who have never masturbated, and is incident to early healthful manhood; that their premature ejaculation is due to excessive stimulation of the passions and their anxiety to complete the sexual act, occurring most frequently in newly married men and those practicing illicit sexual intercourse; and that their impotence is not a pathological condition, is due wholly to the influence of the mind and can be corrected by themselves through exercise of the will-power.

The patients require little treatment, but must be dealt with plainly, and if they can be made to see that you are honest with them, and you can get their confidence, the battle is won. But some are not so easily convinced, and it is often necessary to administer a "placebo" purporting to have a powerful effect, which will generally accomplish the end desired.

However, every patient who comes to you complaining of night emissions, premature ejaculation and impotence cannot be styled as belonging to this class of patients. This should be carefully looked into. If he has ever masturbated, it must be ascertained to what extent it has been carried, the patient must be closely questioned as to gonorrhœa and syphilis, and all statements duly weighed and accepted with proper reserve, and the mind of the physician fully satisfied as to the complaints of the patient being wholly without foundation—a thorough examination being generally deemed advisable.

Nocturnal seminal emissions are usually met with in young men between the ages of sixteen and twenty-five. At this period the sexual functions are most active, and the secretion of semen is continually going on, and must give itself vent in either one way or another. It is, of course, governed to a greater or less degree by the habits of the individual, and will depend somewhat upon the purity of his thoughts, and whether the sexual desires have ever been excited by masturbation, illicit sexual congress or the marriage relation. But to find a healthy young man of ordinary intelligence, living continently, and surrounded by the ordinary environments, and pursuing the usual walks of life, who does not have an occasional nocturnal emission, is an exceedingly rare instance. Usually these emissions are found to occur once a month or once a fortnight, but they may occur two or three times a week without any detrimental results to follow; again, the patient may, for several weeks, be entirely free from them, when they will appear upon several successive nights or even upon the same night.

Bumstead says: In ninety-nine cases out of one hundred, these emissions require no medical or surgical treatment. The chief danger from them lies in the patient attaching undue importance to them, in dwelling upon them, and in making himself miserable over them. If he can be induced to give his mind and body pure thoughts and healthy exercise, and to look upon their occurrence as a physical necessity, nature will take care of the rest.

As to masturbation being the most frequent cause of nocturnal emissions, I wish here to quote the words of Sir James Paget[1]: You may teach positively that masturbation does neither more nor less harm than sexual intercourse practiced with the same frequency, in the same conditions of general health and circumstances. Practiced frequently by the very young—that is, at any time before or at the beginning of puberty—masturbation is very likely to produce exhaustion, effeminacy, over-sensitiveness and nervousness; just as

1 Clinical Lectures and Essays, London, 1875.

equally frequent copulation at the same age would probably produce them, or practiced every day or many times in one day, at any age, either masturbation or copulation is likely to produce similar mischiefs or greater. And the mischiefs are especially likely or nearly sure to happen, and to be greatest, if the excesses are practiced by those who, by inheritance or circumstances, are liable to any nervous disease—spinal irritation, epilepsy, insanity or any other. But the mischiefs are due to the quantity, not to the method of the excesses; and the quantity is to be estimated in relation to age and the power of the nervous system. I have seen as numerous and as great evils consequent on excessive sexual intercourse, as on excessive masturbation; but I have not seen or heard anything to make me believe that occasional masturbation has any other effect on one who practices it than has sexual intercourse, nor anything justifying the dread with which sexual hypochondriacs regard the having occasionally practiced it. I wish I could say something worse of so nasty a practice; an uncleanliness, a filthiness forbidden by God, an unmanliness despised by men.

Marriage has been advised, whenever practicable, for young men of this class, and while it usually accomplishes all that is intended it should, physicians should be very chary in urging this point, and is admissible only when it is absolutely certain that there exists those conditions and circumstances which combine to make a happy marriage, and is not simply a union having for its purpose sexual intercourse, for marriage under these conditions is likely to entail far greater evils than can ever result from nocturnal emissions; nor is illicit sexual congress ever to be advised in lieu of marriage, because of the fact that it invariably leads to excesses which have an effect entirely at variance with the end desired, and leaves the patient in a far worse condition than he was originally.

Another very frequent complaint of this class of patients is a too speedy ejaculation in the effort at coition, which may take place in attempting intercourse with any woman, or with some one woman in particular, especially if the attempt be the first one with her. Here the mind is at fault; over-anxiety to perform the act well is very apt to its being done badly. Young husbands in their eagerness to consummate the rite, not infrequently fail, and I fancy that there are few men who did not ejaculate prematurely when they had connection for the first time.

In January, 1892, I was consulted by a young man, H. E. L., age 22, book-keeper, for premature ejaculation of semen. Came to me in great distress. Had been married four days, and upon first attempt at sexual intercourse, had failed utterly because of ejaculation of semen before intromission could be accomplished. Had not since made effort at repetition of act, fearing he would again fail, and dreading further mortification. Found he had never masturbated, had never had gonorrhœa, nor had he ever been so troubled before in attempting intercourse with other women. I explained to him that his failure was most probably due to normal impetuosity and want of self-control, telling him that it was frequently the case in newly-married men and that he would not likely have any further trouble upon a second effort. On the way to my office next morning, I chanced to meet him, when he greeted me with a happy, confident smile and passed on.

Impotence from the restraining or inhibitory control of the brain over the genito-spinal centre is a condition often met with. That erection may fail or cease under the influence of excitement, depressing or other emotions, or mental preoccupation, is a fact with which every one is familiar.

Thus, it will be found in the history of nearly all cases of rape, that where a considerable struggle has taken place, the performance of the act has been more or less imperfect, due to the muscular exertion, the excited state of the mind and the difficulty attendant upon its performance. Young husbands who were previously potent, and had never indulged in sexual or unnatural excesses, or young men about to have illicit sexual intercourse with some young woman for the first time, are liable to be troubled in this way, the undue stimulation of the passions at their first efforts at copulation having the effect of causing the erection to cease before the act is completed, or even before intromission. As in premature ejaculation, repetition of the effort generally corrects the trouble.

The satisfactory accomplishment of the sexual act can be influenced by the most absurd fancy or the merest whim. One man will be told by a friend of his sexual weakness, and with this story in his mind, he too, will find himself unable for a time to complete the act. The utterance of a vulgar expression, or a coarse word by a woman, may take away all desire for her, and the power and desire yet remain for others. Excessive desire, with gratification long delayed, may temporarily deprive a man of his power.

One of the most frequent causes of impotence, is the fear that coitus is impossible, and you will be told by patients that the moment intercourse is attempted, no matter who the woman may be, or how passionately fond he may be of her, the power leaves him entirely, although the desire is excessive.

Grimaud de Caux[2] relates the instance of a mathematician in whom erection ceased before ejaculation, because his thoughts wandered to the solution of an intricate problem. The wife resorted to the stratagem of partially intoxicating her husband before the attempt at connection, which rendered his powers all that could be desired.

Ribaud[3] relates the story of a young Frenchman who was given the initiatory steps into the pleasures of Venus by a young woman occupying the position of governess in some one of the neighboring country homes, who was a blonde, and always wore when she met him, English boots, corsage and blue silk dress. After a time it became desirable by his family, that a marriage should be consummated by him, but he found himself impotent except under the conditions that the woman be a blonde, she must wear English boots, corsage and blue silk dress, under which circumstances, his powers were vigorous enough. Ribaud, under pretense of giving him a powerful drug which would certainly cure him, administered a "placebo" which brought about the desired result.

Onimus and Legros[4] speak of a young man who was impo-

---

2 Physiologie de l'Espece.

3 Traite de l'Impuissance et de la Sterilite.

4 Traite d'Electricite Medicale.

tent for years after having been surprised at the moment of connection by the husband.

I mention these instances to illustrate to what extent a man's powers may be governed by his mental condition, and the importance of obtaining the confidence of patients who apply afflicted with these imaginary troubles.

